# Disparities in modern health service utilization across socio-demographic and economic inequalities among households in Gida Ayana district, Oromia Regional state, Ethiopia: a community-based cross-sectional study

**DOI:** 10.1186/s12913-023-09527-z

**Published:** 2023-06-08

**Authors:** Edosa Tesfaye Geta, Abebe Wakjira, Wase Benti Hailu

**Affiliations:** 1 Department of Public Health, Institute of Health Science, Wollega University, Nekemte, Ethiopia; 2 Gida Ayana hospital, East Wollega zone, Ayana, Oromia Regional State Ethiopia

**Keywords:** Health care utilization, Households, Socio-economic inequality, Health care disparity

## Abstract

**Background:**

Health care disparities (HCD) occur across a broad range of dimensions and achieving equity in health care is a strenuous task. To overcome the disparities, countries worldwide have started implementing varies policies. HCD remains a challenge in the health care system of Ethiopia. Hence, the study aimed to estimate the disparities in health care utilization (HCU) among households.

**Methods:**

A community-based cross-sectional study was conducted from February 01 to April 30, 2022, among households in Gida Ayana District, Ethiopia. A single population proportion formula was used to determine the 393sample size, and participants were selected using systematic sampling. Data was entered into Epi-data 4.6 and exported to SPSS 25 for analysis. Descriptive analysis and binary and multivariable logistic regressions were performed.

**Results:**

Of the 356 households that participated in the study, 321 (90.2%) of them reported at least one member of their family perceived morbidity in the last six months. The overall level of HCU determined was 207(64.5%), 95% confidence interval (CI),59.0-69.7%. Urban residents (AOR = 3.68, 95% CI = 1.94–6.97), attending secondary school and above (AOR = 2.79, CI = 1.27–5.98), rich (AOR = 2.47, CI = 1.03–5.92), small families (AOR = 2.83, CI = 1.26–6.55), and insured (AOR = 4.27, CI = 2.36–7.71) significantly contributed to HCD.

**Conclusions:**

Households’ overall level of HCU for perceived morbidity was moderate. However, significant disparities were observed in HCU across place of residence, wealth status, level of education, family size, and health insurance. Hence, strengthening the strategy of financial protection by implementing health insurance that focuses on the socio-demographic and economic status of households is recommended to reduce the disparities.

## Background

The goal of universal health coverage (UHC) is to ensure that all people have access to affordable and quality health care, regardless of their economic status, gender, or other characteristics. However, disparities in UHC do exist both between and within countries [1]. Health care utilization (HCU) is the quantification or description of people’s use of health services for the purpose of preventing and curing health-related problems, as well as promoting health and well-being. It may include obtaining information about one’s health status and prognosis [2].

HCU is primarily determined by the need for health services and their availability, as well as the resources available for providing and paying for health services. In countries with few freely available health services, economic status plays a larger role in determining utilization [3].

In health care disparities (HCD), certain groups experience disproportionately poor access to affordable health care. This includes a lack of health insurance as well as poor access to providers or transportation, and these populations also experience disparities in treatment, quality of care, and health outcomes [4]. Not only that, but health is unevenly distributed according to socioeconomic status. Persons of lower income, education, or occupational status experience worse health and die earlier than do their better-off counterparts [5]. The persistence of HCD in many countries indicates that increases in health care coverage and access through affordability alone may not translate into equitable increases in the utilization of health services for all patients. The factors that contribute to health outcomes are complex and involve both economic and social factors [6].

Various studies revealed various types of barriers that exist between patients and services when mapping out the factors of HCU. There are as many categorizations and variations in terminology as there are studies, but they tend to fall under the divisions of geographical, social, economic, cultural, and organizational factors. Accordingly, geographical, socio-economic, and culturally related factors are user-related factors, which the current study aimed to assess, and organizational factors are service-related factors [7, 8].

Poor HCU is a major contributing factor to increased morbidity and mortality in low and middle-income countries (LMICs). In Sub-Saharan Africa (SSA) countries, the percentage of people seeking health care was low, as reported in Mongolia (44.1%), Congo (54.6%), and Ethiopia (38.7%) [9]. Despite the high burden of preventable and curable diseases in LMICs, there is a considerable unmet need for health care [10]. Service availability is still limited, and numerous barriers to access exist, preventing service use, especially for the poorer socio-economic groups [11].

Even though Ethiopia has been implementing the primary health care approach since the mid-1970s, the country quietly continued with a basic challenge comprised of insufficient coverage of services, disproportionate access, inadequate quality of care, and high out-of-pocket expenditure with low health service utilization [12]. Ethiopia is Africa’s second-largest country in terms of population size. However, the country ranks low in access to modern healthcare services compared to other African countries [13]. In 2015, the average outpatient department visit rate in the country was 0.48 visits per person per year. However, the target was two visits per person per year by 2020 [14], which was low in comparison to the world’s 130 countries. The global outpatient age-standardized utilization rate was 5.4 visits per individual per year [15], and the World Health Organization (WHO) recommends around 3 to 4 outpatient visits per person per year [16].

An abundance of studies focused on supplier-side factors determining access rather than demand-side factors that characterize households. Given these premises, identifying the demand-side determinants of modern health services (MHS) utilization contributes to inequalities in health and health care. While there have been improvements in health care coverage in Ethiopia, socio-demographic and economic inequalities remain the driving factors in the utilization of health care. Hence, the study aimed to estimate the disparities in health care utilization across socio-demographic and economic inequalities among households.

## Methods and materials

### Study settings

This study was conducted from February 1, 2022, to April 30, 2022, in the Gida Ayana district. The district is located in the East Wollega Zone, Oromia Regional State, Ethiopia, and is found in the west direction. It is located at (90° 52°N,) and (42° 37°E) at a distance of 430 km from Addis Ababa, the capital city of the country. The total catchment area of the district was about 183,063m^2^. Its total population was estimated to be 135,980, of which 69,350 were females and 66,630 were males. In the district, there were about 30,357 households. The district had 1 general hospital, 5 health centers, 29 health posts, and 26 private clinics.

### Study design and population

A community-based, cross-sectional study was used. All households in Gida Ayana district were considered the source population, and systematically selected households were considered the study population, whereas the selected head of household was considered the study unit.

### Eligibility criteria

Household heads or family representatives who were greater than or equal to 18 years of age and who had resided in the area for more than six months were included in the study, whereas household heads who were government employees and unavailable during the study period were excluded from the study.

### Sample size determination and sampling techniques

The required sample size for the study was determined by using formula of single population proportion (P) which was 58.4% of the households sought and utilized health care from modern health facilities in Ethiopia [17] and using 95% CI with 5% margin of error and 5% non-response rate; Zα/2 = critical value for normal distribution at 95% confidence interval which equals to 1.96 (value at α = 0.05) adding 5% of non-response rate;

n= (Zα/2)^2^*P(1-P/d^2^

n= [(1.96)^2^*0.584(1-0.584)]/(0.05)^2^ =393

**Fig. 1:**
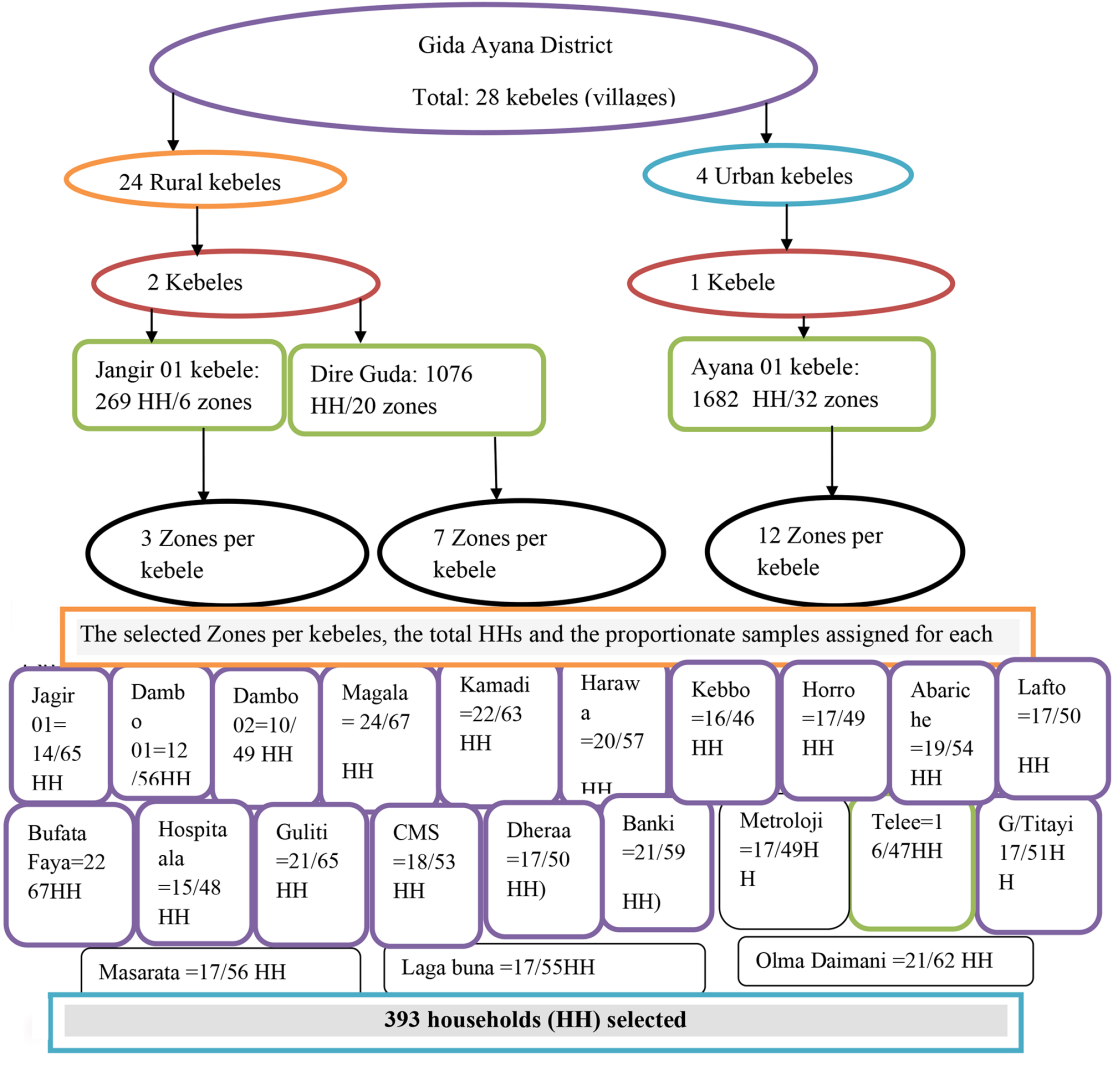
Sampling technique to select study households in Gida Ayana district, 2022

All lists of 28 kebeles (villages) in the district were taken from the administrative office of the district. First, 3 kebeles were selected randomly by lottery method. In the second stage, 22 zones based on its proportion to each Kebele were randomly selected. Finally, the required sample size for the selected zones determined using the population proportionate to the sample size (PPS) and systematic random sampling was used to select the study subjects in each of the selected zones. Because of difference in numbers of households, “K^th^” was calculated separately for each zone and by dividing the total number households in each zone (S) to the corresponding sample size (s). The number “K” obtained by dividing S/s was used to identify the interval among selected households from each zone. Since, the sampling fraction was ‘K’, every K^th^ household was included in the study and to select the first household from 1 to K, lottery method was used. One respondent per household was interviewed. If there was more than one eligible respondent in the compound, the head of household was selected to be interviewed (Fig. 1).

### Data collection tool and procedures

Semi-structured questionnaires were developed after reviewing different literature. All tools were prepared in English language and translated to Afan Oromo language for interview. Data was collected by face-to-face interviews with household heads.

### Study variables

Modern health service utilization was measured as a dependent variable, and the independent variables were household head-related factors (age, sex, religion, marital status, occupation, education, family size, wealth status, and resident) and travel time to the modern health facility.

### Operational definitions

#### Modern health services:

Health services which were provided by licensed health institutions including public and private health care facilities (hospitals, health centers, clinics, health posts and private non-profit organizations) [18].

#### Modern health service utilization:

Utilization of health service was measured as the number of utilizing the services (diagnostic or treatment) from modern health facilities [2], made by at least one household member at least once in the previous 6 months. It was binary dichotomous variable measuring health service utilization, coded as ‘1’ (Utilized health care) and ‘0’ (Did not utilize health care) based on the question, *did you/your families visit modern health facilities for health care in the last six months*?

#### Household wealth index:

Households’ assets data was collected on the kinds assets they own. Then factor scores were derived using principal component analysis (PCA), and then the composite scores were categorized into two tiles. The 1st 50% tile was classified as; 1 = **Poo**r, whereas 2nd 50% tile was considered as; **2 = Rich**.

### Data quality management

To ensure the quality of the data, data collectors and supervisors received two days of training on the objectives, methodology, sampling technique, ethical issues, data collection instrument, and data collection procedures. Data was collected by five experienced health professionals who had a bachelor’s degree and two supervisors who had a master’s degree in health. After discussion and a common understanding of the data collection tool, there was regular cross-checking by the data collectors for the completeness of the questionnaires, and the data collectors strictly followed the data collection procedures.

A pre-test was conducted on 20 (5%) of the calculated sample size in another kebele of Gida Ayana District. During the data collection period, close supervision and monitoring were done by the team to ensure the quality of the data. The completeness and consistency were checked in the field by the data collectors.

### Data analysis and presentation

Data was cleaned and entered into Epi-data Manager 4.6 before being exported to SPSS 25 for analysis. Descriptive statistics were computed and presented using frequencies, proportions, summary statistics, graphs, and tables. For a finally fitted multivariable logistic regression model, model fitness was checked by Hosmer-Lemeshow goodness-of-fit and the P-value was found to be 0.910. Initially, a binary logistic regression analysis was computed to identify the significant effect of each independent variable on HCU, and then to identify potential candidate variables at P<0.25 for the final model, a multivariable logistic regression was conducted to determine the effects of socio-demographic and economic factors on the probability of modern HCU among households. The final p-value of < 0.05 was used to declare the significant factors, along with the odds ratio (OR) and 95% confidence interval (CI).

## Results

### Socio-demographic and economic characteristics

A total of 356 household heads participated in the study, with a response rate of 90.6%. The average age of the respondents was 38.21 ± 8.5 years. Heads of the household were predominantly male (312, 87.6%) and married (333, 93.5%).

Regarding the place of residence, the majority of them 230(66.4%) were urban residents. More than half of the household heads 225(63.2%) were farmers and their wealth status was computed using principal component analysis (PCA) which majority of them 277(77.8%) were relatively poor. Regarding the family size, the majority of the households 254(71.3%) had minimum of five family members and 153(43%) of the participants had no formal educations that were unable to read and write (Table 1).

**Table 1:** Socio-demographic and economic characteristics of households in Gida Ayana District, Oromia Regional State, Ethiopia, 2022

Variables (n = 321)	Response category	Health care utilization		
		Yes (%)	No (%)	Total (%)
Place of Residence	Urban	151(47)	50(15.6)	201(62.6)
	Rural	56(17.4)	64(19.9)	(37.4)
Age in years	20–29	46(14.3)	17(5.3)	63(19.6)
	30–39	92(44.4)	41(36)	133(41.4)
	40–49	48(15)	38(11.8)	86(26.8)
	50 above	21(6.5)	18(5.6)	39(12.1)
Sex	Male	170 (53)	110(34.3)	280(87.2)
	Female	37(11.5)	4(1.2)	41(12.8)
Religion	Orthodox	56(17.4)	29(9)	85(26.5)
	Muslim	43(13.4)	27(8.4)	70(36.4)
	Protestant	74(23.1)	43(13.4)	117(36.5)
	Wakefata	34(10.6)	15(4.7)	49(15.3)
Marital status	Married	194(60.4)	105(32.7)	299(93.1)
	Divorced/ widowed	13(4)	9(2.8)	22(6.9)
Wealth status	Poor	159(49.5)	101(31.5)	260(81)
	Rich	48(15)	13(4)	61(19)
Occupation	Farmer	121 (37.7)	85(26.5)	206 (64.2)
	Merchant	86(9)	29(26.8)	115(35.8)
Education level	No formal	80(24.9)	67(20.9)	147(45.8)
	Primary	48(15)	28(8.7)	76(23.7)
	Secondary & above	79(24.6)	19(5.9)	98(30.5)
Family size	< 5 members	72(22.4)	14(4.4)	86(26.8)
	≥ 5 members	135(42.1)	100(31.2)	235(73.2)
Travel time to modern health facility	<1 hour	126(39.3)	43(13.4)	169(52.6)
	≥1 hours	81(25.2)	71(22.1)	152(47.4)

### Modern health services utilization

**Fig. 2:**
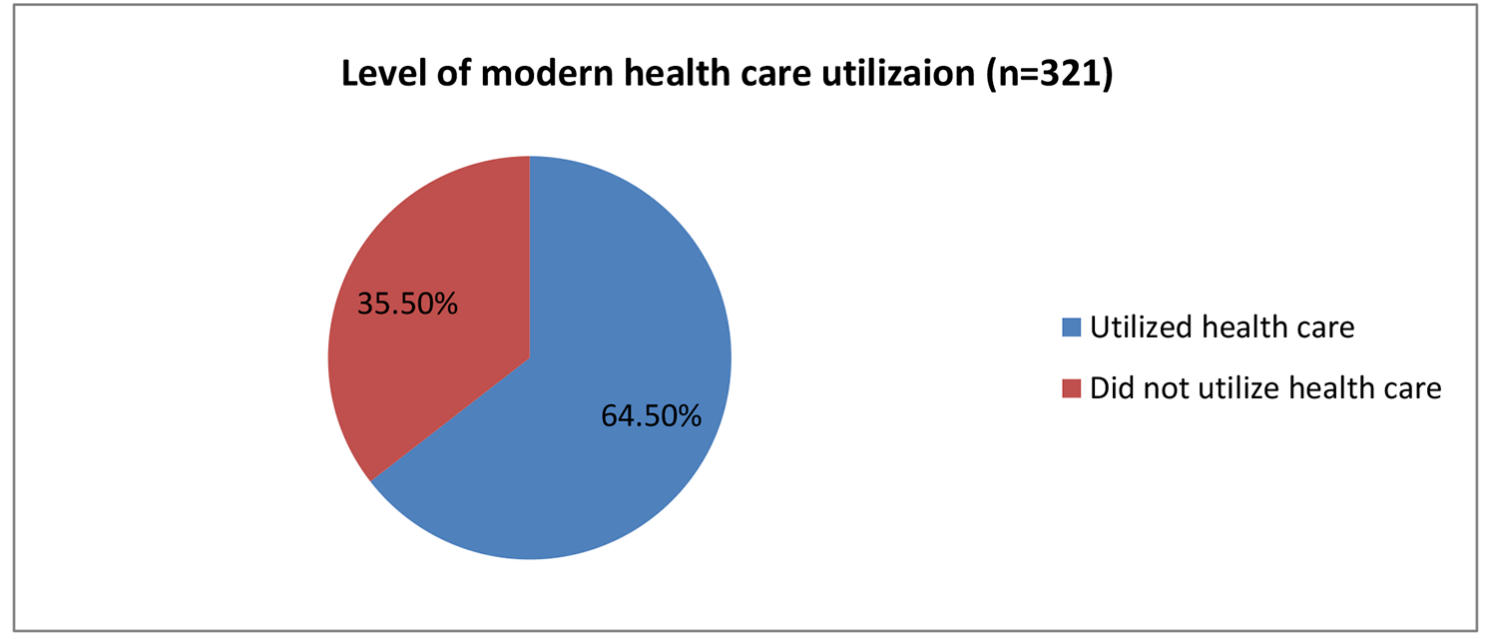
Level of modern health care utilization among households in Gida Ayana District, Oromia Regional State, Ethiopia, 2022

From a total of 356 household heads participated in the study, 321(90.2%, 95% CI = 86.6–93.3%)] of them had reported perceived morbidity in which at least one member of their family fell in the last six months. Among participants who reported their family perceived morbidity 266(74.7%) sought health, out of which only 207(64.5%, 95% CI = 59-69.7%) of them utilized modern health care by visiting modern health facilities (Fig. 2).

The level of modern HCU was also estimated across the households’ socio-demographic and economic inequalities. Accordingly, all most all (90.2%, 95% CI = 76.9–97.3%) female household heads utilized health care and only (60.7%, 95%=54.7–66.5%) of male household heads utilized health care for their family perceived morbidity. Regarding the place of residence, the three fourth of the urban resident households utilized health care (75.1%, 95% CI = 68.6–80.9%) whereas less than half of the rural residents utilized health care (46.7%, 95% CI = 37.5–56%). Similarly, more than the three fourth of relatively rich households utilized health care (78.7%, 95% CI = 66.3–88.1%), and (61.2%, 95% CI = 54.9–67.1%) relatively poor households utilized the care.

Also, the health insurance membership of the households was assessed. Accordingly, the proportion of HCU among insured households was estimated which indicted that more than three fourth of the insured households (79.7%, 95% CI = 72.5–85.8%) and only about half of the uninsured households (50.6%, 95% CI = 42.8–58.4%) utilized the health care for their family members perceived morbidity (Table 2).

**Table 2:** Level of modern HCU across socio-demographic and economic differences among households in Gida Ayana district, Oromia Regional state, Ethiopia, 2022

Variables (n = 321)	Response category	95% CI, Level of HCU (%)
Sex	Male	60.7(54.7–66.5)
	Female	90.2(76.9–97.3)
Place of residence	Urban	75.1(68.6–80.9)
	Rural	46.7(37.5–56)
Age in years	20–29	73(60.3–83.4)
	30–39	69.2(60.6–76.9)
	40–49	53.5(42.2–64.3)
	≥ 50	53.8(37.2–69.9)
Marital status	Married	64.9(59.2–70.3)
	Divorced or widowed	59.1(36.4–79.3)
Wealth status	Poor	61.2(54.9–67.1)
	Rich	78.7(66.3–88.1)
Occupation	Farmer	58.7(51.7–65.5)
	Merchant	74.8(65.8–82.4)
Education level	No formal education	54.8(46.4–63)
	Primary school	80.6(71.4–87.9)
	Secondary school and above	63.2(51.3–73.9)
Family size	< 5 members	83.7(74.2–90.8)
	≥5 members	57.4(50.9–63.9)
Health insurance	Insured	79.7(72.5–85.8)
	Uninsured	50.6(42.8–58.4)

Among those household heads 114(35.5%) who did not seek modern health care by vising modern health facilities, they reasoned out main reasons why they did not seek modern health care. Accordingly, 20(17.5%) of them were recovered from the illness without any treatment, 26(22.8%) of reasoned out there was no modern health facility at nearby their destination to visit, 20(17.5%) of them perceived that the services they need were not available in the nearest health facility, 30(26.3%) of them could not afford the payment to visit modern health facilities, 15(13.2%) perceived that adequate drugs and laboratory services were not available in the facility, and 3(2.6%) of did not seek MHS due to different others reasons.

**Fig. 3:**
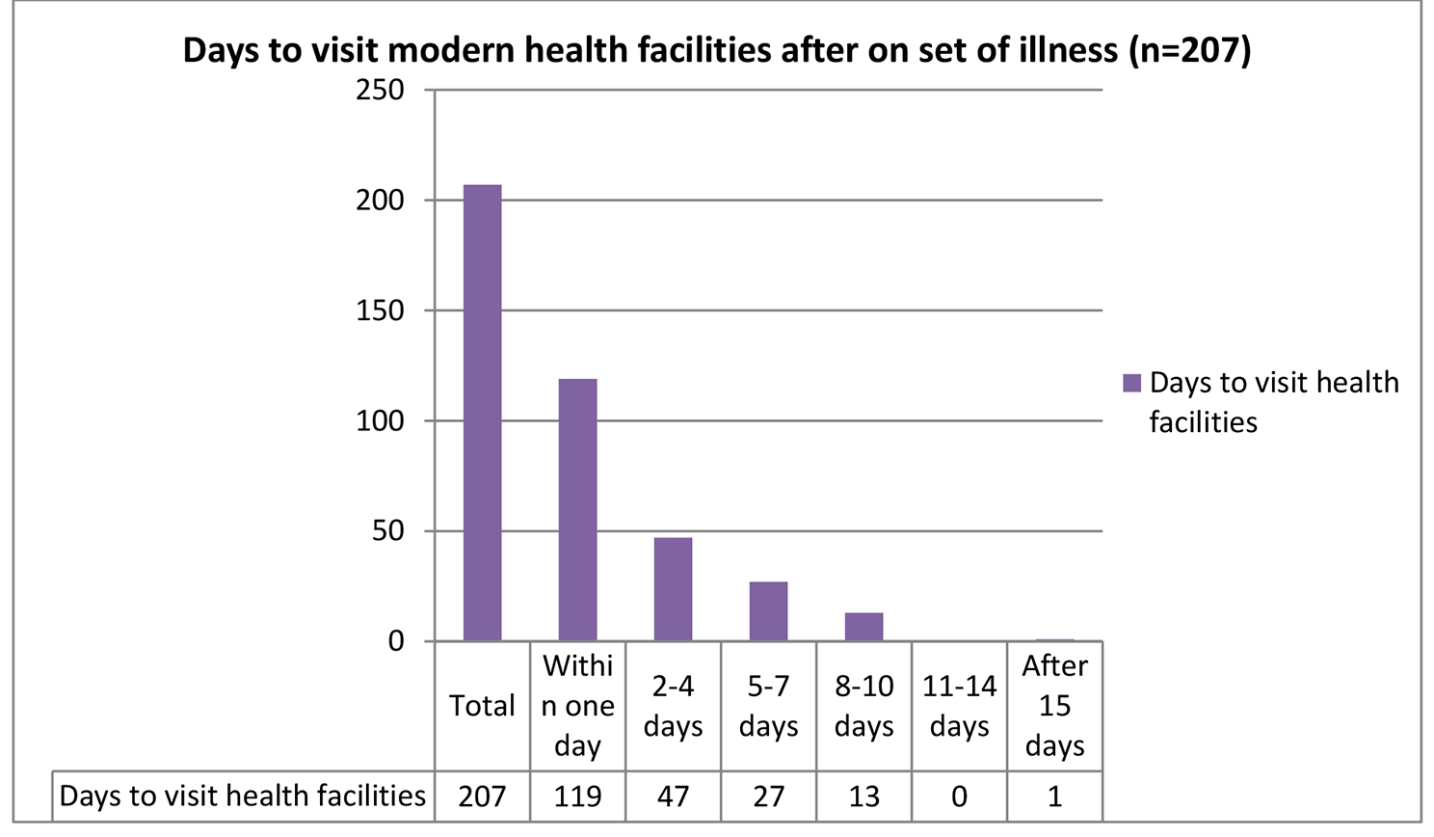
Time in days taken to seek and utilize modern health care of households in Gida Ayana district, Oromia Region, Ethiopia, 2022

Among household heads who sought and utilized modern health care, 132 (63.4%) of them delayed seeking modern HCU, and they visited modern health facilities after seeking health from different institutions. Accordingly, 25 (18.9%) of them visited traditional healers, 55 (41.7%) of them used holy water, 24 (18.2%) of them reported that the illness had subsided for a short time but reoccurred, and 28 (21.2%) of them visited different spiritual healings. More than half of the households 119(57.5%) sought modern health care, visiting modern health facilities within one day of illness onset (Fig. 3).

**Fig. 4:**
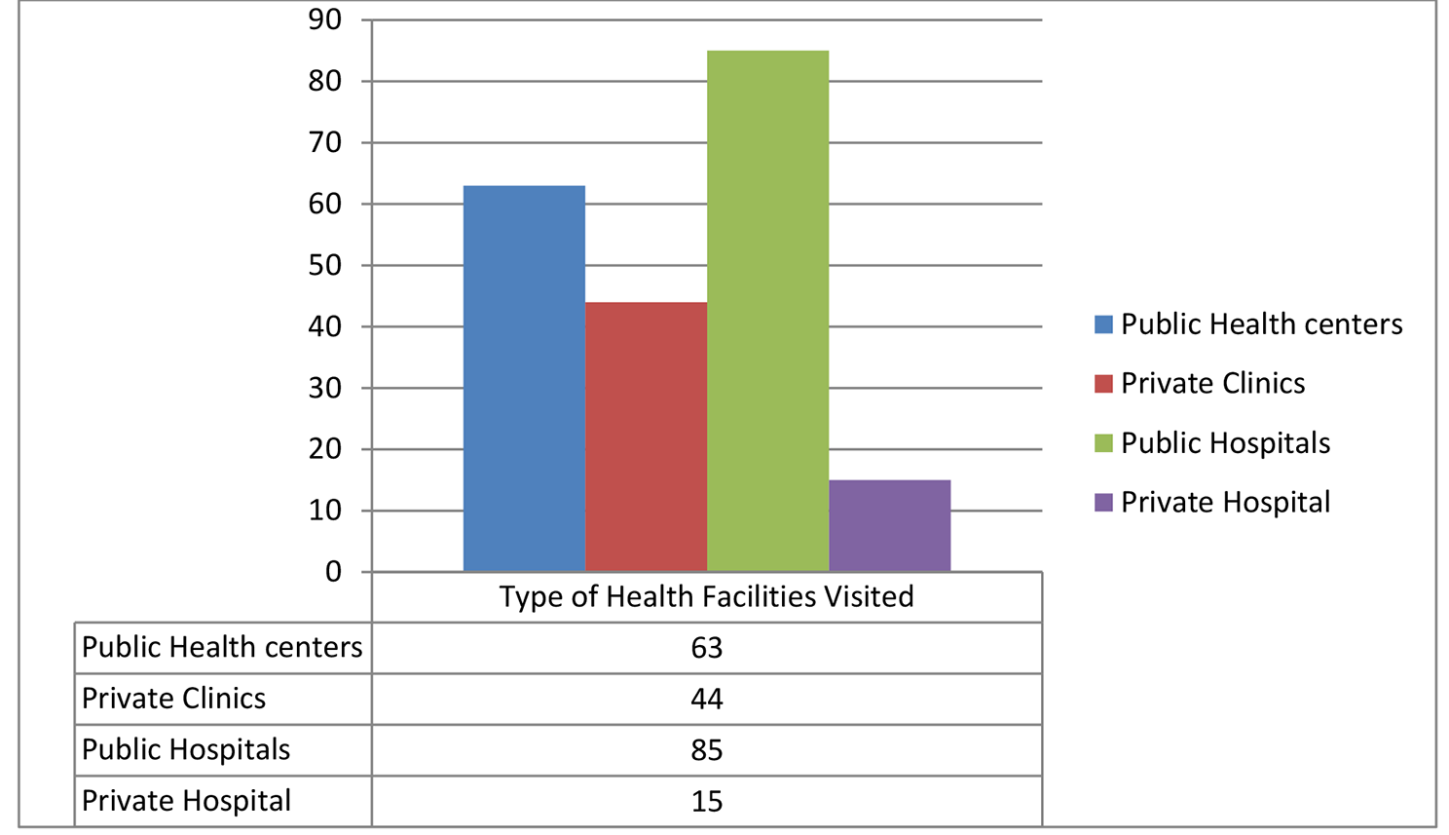
Type of modern health facilities visited by households to utilize health care in Gida Ayana district, Oromia Regional, Ethiopia, 2022

The households whose family members sought modern health care for their perceived morbidity, visited different health facilities, including public and private clinics. The majority of the households 144(54.1%) traveled to the nearest modern health facility for less than one hour to utilize health care (Fig. 4).

The frequency of health facility visits was estimated among households based on their socio-economic and demographic characteristics. For the perceived morbidity of their family, on average households visited health facilities at least two times in past six months (2.33, 95% CI = 2.18–2.49, p = 0.00). Urban households visited modern health facilities 2.42 ± 1.06 times whereas the rural households visited the health facilities 2.11 ± 1.23 times for their family perceived morbidity (Table 3).

**Table 3:** Frequency of modern health facility visits among households in Gida Ayana District, Oromia Regional Sate, Ethiopia, 2022

Variables	Response category	Frequency of modern health facility visits, Mean (± SD)
Sex	Male	2.35(1.11)
	Female	2.24(1.14)
Place of residence	Urban	2.42(1.06)
	Rural	2.11(1.23)
Age	20–29	2.48(1.23)
	30–39	2.52(1.02)
	40–49	2.08(1.09)
	50 above	1.76(1.09)
Marital status	Married	2.35(1.12)
	Divorced or widowed	2.15(0.99)
Wealth status	Poor	2.40(1.13)
	Rich	2.10(1.06)
Occupation	Farmer	2.27(1.16)
	Merchant	2.42(1.06)
Educational status	No formal education	2.38(1.13)
	Primary school	2.04(1.05)
	Secondary school and above	2.47(1.12)
Family size	< 5 members	2.46(1.07)
	≥ 5 members	2.27(1.13)
Health insurance	Insured	2.95(1.03)
	Uninsured	1.45(0.50)

**Note**: *SD = standard deviation*

### Predictors of modern health service utilization

The study calculated the effects of socioeconomic and demographic factors on disparities in MHS utilization. Accordingly, urban resident households were 3.7 times more likely to utilize health services (AOR = 4.52, 95% CI = 1.61–12.73) compared to rural resident households.

Households that were relatively poor were 67% less likely to utilize modern health care compared to the households who were relatively rich (AOR = 0.33, 95% CI = 0.15–0.70). In addition, family size was identified as a significant variable to affect the utilization of health services, and the study revealed that households with fewer than five family members were 2.5 times more likely to utilize health services (AOR = 2.52, 95% CI = 1.15–5.51) compared to households with a minimum of five family members. The health insurance status of the households was also assessed. Accordingly, the insured households were 4.3 times more likely to utilize health services (AOR = 4.27, 95% CI = 2.36–7.71) compared to uninsured households (Table 4).

**Table 4:** Predictors of MHS utilization among households in Gida Ayana District, Oromia Regional State, Ethiopia, 2022

Variables (n = 321)	Response Category	Health Service Utilization			Odds ratio and 95% CI	
		*Yes (%)*	*No (%)*	*Total (%)*	*COR*	*AOR*
Place of Residence	Urban	151(47)	50(15.6)	201(62.6)	3.45(2.14–5.58)	4.52(1.61–12.73) **
	Rural	56(17.4)	64(19.9)	120(37.4)	1	1
Age in years	20–29	46(14.3)	17(5.3)	63(19.6)	2.32(1.00-5.37)	1.29(0.45–3.71)
	30–39	92(44.4)	41(36)	133(41.4)	1.92(0.93–3.98)	1.34(0.57–3.17)
	40–49	48(15)	38(11.8)	86(26.8)	1.08(0.51–2.32)	1.48(0.62–3.54)
	50 above	21(6.5)	18(5.6)	39(12.1)	1	1
Wealth status	Poor	159(49.5)	101(31.5)	260(81)	0.43(0.22–0.83	0.33(0.15 − 0.70) **
	Rich	48(15)	13(4)	61(19)	1	1
Occupation	Farmer	125 (37.7)	81(26.5)	206(64.2)	0.48(0.29–0.80)	0.79(0.41–1.54)
	Merchant	86(9)	29(26.8)	115(35.8)	1	1
Education	No formal	80(24.9)	67(20.9)	147(45.8)	0.29(0.16–0.52)	0.34(0.17–0.68) **
	Primary	48(15)	28(8.7)	76(23.7)	0.41(0.21–0.82)	0.56(0.25–1.22)
	Secondary & above	79(24.6)	19(5.9)	98(30.5)	1	1
Family size	< 5 members	72(22.4)	14(4.4)	86(26.8)	3.81(2.03–7.14)	2.52(1.15–5.51) *
	≥ 5 members	135(42.1)	100(31.2)	235(73.2)	1	1
Travel to health facility	< 1 hour	126(39.3)	43(13.4)	169(52.6)	2.57(1.61–4.11)	0.86(0.30–2.44)
	≥ 1 hours	81(25.2)	71(22.1)	152(47.4)	1	1
Health insurance	Insured	122(38)	31(9.7)	153(47.7)	3.84(2.34–6.31)	4.27(2.36–7.71) ***
	Non-insured	85(26.5)	83(25.9)	168(52.3)	1	1

***Notes***: *=P-value < 0.05, **=P-value < 0.01, ***=P-value < 0.001 and 1 = reference, CI = Confidence interval, COR = Crude odds ratio, AOR = Adjusted odds ratio

## Discussion

The current study estimated the level of modern HCU for perceived morbidity and the disparities in health care utilization across the socio-demographic and economic factors among households. Accordingly, the overall level of health care utilization for the perceived morbidity was 64.5%, which varied based on the socio-demographic and economic status of the households. The study revealed that there was a significant difference in modern health care utilization across the socio-demographic and economic status of the households. The overall level of health care utilization was higher compared to the study findings in Ethiopia [17]. However, the level and odds of health care utilization significantly varied across the socio-demographic and economic differences among households.

The study showed that those households that were relatively poor were 67% less likely to utilize health care compared to rich households because of their perceived morbidity. This was also explained by the HCU levels in urban and rural resident households, which were 78.7% and 61.2%, respectively, when a family member became ill. This finding was consistent with the findings of study result in Ethiopia, which found that higher-income households were more likely to use health care than lower-income households [17–20].

In addition, the study also identified that the place of residence was a significant factor that contributed to the disparity in HCU; urban households were at least four times more likely to utilize health care from modern health facilities compared to rural residents. This is supported by the fact that the level of HCU among urban residents was 75.1%, whereas it was 46.7% among rural households. Similarly, the study conducted in Burkina Faso showed that those households that lived in urban areas were almost twice as likely to utilize health care compared to rural residents [21].

In addition, a study in Greece found that the urban population was more likely to use health services than the rural population [22], and a study in Iran found that 58% of the urban population and 42% of the rural population sought and used health care, as well as a study in Wales that found rural households were less likely to visit modern health facilities than urban households [23]. This could be due to the fact that in urban areas, there could be a greater number of modern health facilities that are easily accessible, which could enable urban households to seek out and utilize MHS more likely compared to rural households.

The study also revealed that a lack of formal education significantly contributed to disparities in the HCU when members of a household’s family perceived illness and sought modern health care. In this study, household heads with a secondary education or higher were nearly three times more likely to use modern health care than those with no formal education. Similarly, a significant difference was observed in the level of health care utilization among households based on their level of education. Accordingly, the level of health care utilization among households that had no formal education, attended primary education, or secondary education or above was 54.8%, 80.6%, and 63.2%, respectively. It was similar to the findings of a study in Nigeria, which found that education beyond the senior secondary school certificate of the mother household head was associated with being 5.3 times more likely to seek health within 24 hours of the onset of illness [24], and a study in Greece found that primary education was associated with more visits to health facilities [22]. This could be due to when the household heads are educated; they could have adequate information about their family member’s health problems and the health service availability in modern health facilities that could enable them to seek and utilize modern health care for their perceived morbidity.

Another study conducted in Nigeria also showed that the household head’s educational level and income significantly predicted increased health care seeking and utilization; high education and high family socioeconomic status were strong predictors of early care-seeking and care-seeking outside the home [24].

Again, the present study showed that those households with less than five members were almost three times more likely to utilize health services compared to those with a minimum of five family members, and the level of HCU was higher among households with a small family size compared to those with a large family size. In this case, the level of the HCU was estimated to be 83.7% and 57.4% among households with less than five family members and households with at least five family members, respectively. It was in line with the study conducted in Nigeria, which revealed that parents who had only one child were eight times more likely to seek health care within 24 hours of the onset of illness [24]. This could be because households with large families spend more money on other basic needs to support their families rather than on health care.

Currently, the government of Ethiopia is implementing community-based health insurances to improve the HCU among informal employed households. Hence, the current study estimated the odds and level of HCU among insured and uninsured households. Accordingly, the insured households were at least four times more likely to utilize health services compared to the uninsured households. This is explained by the fact that the level of HCU was significantly higher among insured households (79.7%), whereas only half of the uninsured households (50.6%) were able to utilize health care. Similarly, the study conducted in America showed that individuals with health insurance had 2.4 higher odds of using outpatient care than individuals who lacked insurance [25], and the study in South Africa showed that households with medical insurance were five times more likely to utilize health services [26].

Despite the fact that the study accurately estimated the effects of socio-demographic and economic inequalities on modern health care utilization for perceived morbidity among households, the study may have limitations due to recall bias in the frequency of health facility visits and which type of modern health facilities the households used for the illnesses in which the households’ family members fell ill in the previous six months.

## Conclusions

Households’ overall utilization of modern health care for perceived morbidity was moderate. However, significant disparities in the utilization of health care across differences in place of residence, educational level, family size, wealth status, and health insurance membership among households were observed. As a result, strengthening the financial protection strategy through the implementation of health insurance that focuses on socio-demographic and economic factors among households is strongly recommended in order to improve modern health care utilization and its disparities.

## Data Availability

The datasets used and/or analyzed during this study are available from the corresponding author on reasonable request.
